# Are we aware of stroke risk factors and warning signs amid COVID-19? Knowledge and attitude towards risk-factors, signs and symptoms of stroke among the UAE population during COVID-19 pandemic: A cross-sectional study

**DOI:** 10.12688/f1000research.129873.1

**Published:** 2023-03-23

**Authors:** Azza Ramadan, Zelal Kharaba, Rose Ghemrawi, Asim Ahmed Elnour, Nadia Hussain, Parisa Kouhgard, Nosayba Al-Damook, Shymaa Abou Hait, Lena Al Ghanem, Rawan Atassi, Ranem Chkh Sobeh, Ahmad Z. Al Meslamani

**Affiliations:** 1 AAU Health and Biomedical Research Center, Al Ain University, Abu Dhabi, United Arab Emirates; 2 Pharmaceutical Sciences, Al Ain University, Abu Dhabi, United Arab Emirates; 3 Program of Clinical Pharmacy, Al Ain University, Abu Dhbai, United Arab Emirates; 4 Honorary Associate Lecturer, Faculty of Medical Sciences, Newcastle University, Newcastle, UK; 5 Cleveland Clinic, Cleveland Hospital, Abu Dhabi, United Arab Emirates

**Keywords:** Knowledge, Attitude, Awareness, Cross-sectional, Stroke, Survey

## Abstract

**Background**: Despite significant advancements in healthcare, the burden of stroke continues to rise in the developed world, especially during the COVID-19 pandemic. Association between COVID-19 infection and stroke is well established. Factors identified for the delay in presentation and management include a lack of awareness regarding stroke. We aimed to assess the general public knowledge and attitudes on stroke and stroke risk factors in the United Arab Emirates during the COVID-19 pandemic.

**Methods**: A cross-sectional study was conducted between September 2021 and January 2022 among adults≥ 18 years old. Participants completed a self-administered questionnaire on sociodemographic characteristics and stroke knowledge and attitudes. Knowledge and attitude scores were calculated based on the number of correct responses. Linear regression analysis was performed to determine the factors related to knowledge and attitude towards stroke.

**Results**: Of the 500 respondents, 69.4% were females, 53.4% were aged between 18 and 25, and nearly half were students (48.4%). The mean knowledge score was 13.66 (range 2-24). Hypertension (69%), smoking (63.2%), stress (56.4%) obesity/overweight (54.4%), and heart disease (53.6%) were identified as risk factors. Overall, the knowledge of signs/symptoms was suboptimal. The mean attitude score was 4.41 (range, 1-6); 70.2% would call an ambulance if someone were having a stroke. A monthly income of 11,000-50,000 AED and being a student were associated with positive knowledge. Being a non-health worker and lacking access to electronic media sources were associated with worse attitudes.

**Conclusion**: Overall, we identified poor knowledge and suboptimal attitudes toward stroke. These findings reflect the need for effective public health approaches to improve stroke awareness, knowledge, and attitudes for effective prevention in the community. Presently, this is of utmost necessity, given the increased occurrence of stroke and its severity among COVID-19 patients.

## Introduction

According to the World Health Organization, stroke is the ‘incoming epidemic of the 21 ^st^ century’, which is unsurprising considering that it accounted for 12.2 million incident cases, 101 million prevalent cases, 143 million disability-adjusted life-years lost, and 6.6 million deaths in 2019. ^1^ The United Arab Emirates (UAE) was reported to be among the three countries within the Middle East and North Africa region with the highest prevalence of stroke in 2019. ^2^ Alarmingly in 2022, it was reported by the Ministry of Health and prevention that 50% of stroke patients were under the age of 45 as opposed to the global incidence of 80% over the age of 65. ^3^ Despite significant medical advancements, morbidity and mortality from stroke remain high. ^4^^–^^6^ It is associated with a high burden of healthcare costs, upwards of US$ 721 billion estimated in 2017. ^7^ Furthermore, the absolute number of strokes is expected to rise due to the worldwide aging phenomenon.

Knowledge and awareness play an essential part in the early detection of chronic conditions such as stroke. The World Health Organization has recognized the importance of driving efforts toward increasing knowledge about disease conditions and risk factors. Epidemiological studies have shown that higher health literacy positively relates to preventive measures, especially against chronic non-communicable diseases. ^8^^,^^9^ The level of knowledge can affect people’s attitudes and practices; on the other hand, negative attitudes and practices could increase the risk of disease and subsequent morbidity and mortality. Regarding stroke, early identification of symptoms and appropriate and timely management can positively influence individuals’ outcomes. ^10^

Stroke is a multifactorial condition; the most significant risk drivers include hypertension, high body mass index, dyslipidemia, diabetes, smoking, and a family history of stroke. ^11^^,^^12^ Analysis of reports pertaining to the COVID pandemic shed light on the fact that COVID infection raised the risk of stroke by more than two times. ^13^ Also, another study has demonstrated that COVID-19 associated ischaemic strokes tend to be more severe and can lead to death compared to non-infected stroke patients ^14^ and COVID vaccination was associated with both ischemic stroke and vaccine-induced immune thrombotic thrombocytopenia ^15^; and cerebral venous thrombosis. ^16^

Despite effective therapies for stroke, only a small proportion of patients receive medications due to delayed presentation. ^17^ However, many strokes are preventable, suggesting that prevention strategies should be at the forefront of stroke management with primary and secondary prevention measures that target the risk factors. ^18^ Factors influencing the management of stroke include awareness of stroke signs and symptoms, awareness of the importance of early management, educational level, distance from the hospital, and past history of stroke. ^19^^,^^20^

Identifying baseline stroke knowledge in the general population is crucial to developing effective, targeted, and appropriate health promotion programs for stroke prevention. There is limited data on people’s knowledge and attitudes towards stroke in the UAE. In 2019, a knowledge survey study pertaining to stroke was conducted in Sharjah city, UAE. ^21^ It was reported that most participants had low to average knowledge levels. ^21^ Given the increased risk of stroke in COVID patients and the scarcity of data regarding stroke, we aimed to assess the general public knowledge and attitudes on stroke and stroke risk factors across the entire UAE during the COVID pandemic.

## Methods

### Ethical considerations

The study received the required ethical approval from the research ethics commission (REC) at Al Ain University (AAU-REC-B3, September 2021).

### Study population and sample size

This was a cross-sectional study among 500 subjects ≥ 18 years and from the general public in Abu Dhabi, Dubai, Sharjah, Ajman, and other UAE cities. The study was conducted from September 2021 to January 2022 i.e. during the COVID-19 period. Random/stratified sampling included subjects who fulfilled the inclusion criteria and were willing to participate in this study. The sample size was chosen with an assumed prevalence of hypertension, the most significant risk factor for stroke, 52% ^22^ also via using a Raosoft sample size calculator, ^23^ a confidence limit of 5%, and a 95% confidence interval estimate of the proportion. A minimum sample size of 384 was needed. The study included both locals (Emiratis) and expats (residents).

### Inclusion and exclusion criteria

UAE residents (> 2 years living in the UAE) regardless of their nationality, over the age of 18 years and consented (on the questionnaire by ticking the agreement option) to participate were included in this study. Participants who lived < 2-years in the UAE or did not consent to participate were excluded.

### Design of questionnaire

The questionnaire was developed after a careful literature review of previous studies utilizing standardized and validated instruments ^24^^–^^28^ and expert feedback. The questionnaire was constructed in Arabic and English. The translation process was via an Authentic Medical Translator who was officially approved to translate English to Arabic (the official language of residents in the UAE). Pretesting of the questionnaire was performed to gather information on its feasibility, and assess time to completion, understandability, and consistency. After pretesting, the survey was conducted online, and the responses were collected in an Excel sheet.

Questions evaluating the knowledge of stroke comprised the organ affected, stroke attributes (preventable or recurrent), effect on daily activities, treatment, prevention, risk factors, and signs and symptoms. Questions evaluating attitude (the approach) comprised actions to take if someone showed signs and symptoms of a stroke. Lastly, there was a question about the sources of information. For the 25-item knowledge questions, the score range was 0-25. For attitudes, the score range was 0-6. Each correct statement for knowledge and optimal attitude got a 1 otherwise 0.

### Validation of the study questionnaire

The validation test was conducted for the edited questionnaire version. A questionnaire draft was written and sent to a panel of experts in the pharmacy profession at Al-Ain University to test the content validity of the survey. They examined many factors of the questionnaire, including the length, conciseness, language, clarity, time, appropriateness, and bias of questions. Content validation of a questionnaire was aligned with recommendations. ^24^

### Reliability testing of the study questionnaire

The reliability test was conducted as a pilot study on 50 students to achieve the most acceptable Cronbach’s values. Additionally, preliminary pilot testing was carried out to ensure the understandability and practicality of the questionnaire.

### Data collection

The final version of the study survey was administered online. Participants were briefed about the study’s purpose and informed about the study’s confidentiality and anonymity policy.

### Statistical analysis

Data were verified at the end of the survey and before the analysis. The data analysis was performed using the SAS software (version 9.4 SAS Institute, Cary, NC) (alternative; PSPP software; free open source). Respondent’s socio-demographic characteristics were stated using descriptive statistics. Means, standard deviations (SD), and proportions were generated to describe the overall sample characteristics (age, gender, occupation, marital status, education, income, country of birth, and comorbidities). Multivariable linear regression modeling was applied to determine the variables associated with stroke-related knowledge and attitude. All associations were considered significant at the alpha level of 0.05.

## Results

### Demographic characteristics

Table 1 presents the sociodemographic characteristics of the study population. Of the 500 participants who completed the questionnaire, 69.4) were females, 53.4% were aged between 18 and 25, and 59.4% were single. Among the participants, 79.8% were expats. Additionally, roughly half earned AED **≤**10,000 (Dirham) per month. Among the study participants, 50.8% had no known comorbid, 9.4% had hypertension, 6.6% had diabetes, 3.4% had cardiovascular disease, and 1.8% had a history of a stroke. Regarding the sources of information about stroke, approximately 50.4% and 40.4% of the study participants reported electronic media and friends, respectively. Fewer than one-third stated a healthcare provider as an information resource. Around 15.63% were healthcare students, and 10.03% were healthcare workers. It’s noteworthy to mention that 339 of the 500 participants answered the question that determines if the respondents are either healthcare workers or healthcare students.

**Table 1.** Sociodemographic characteristics of the study participants (n = 500).

Characteristics	Number	Percent
**Gender**		
Female	347	69.40
**Age, years**		
18-25	267	53.40
26-35	93	18.60
36-45	92	18.40
46-55	39	7.80
56-55	6	1.20
65 and above	3	0.60
**Country of birth**		
Africa	9	1.80
Arabic country	221	44.20
Gulf	13	2.60
North America	4	0.80
Others	32	6.40
UAE	221	44.20
**Educational level**		
No formal education	3	0.60
Undergraduate	256	51.20
Graduate	205	41.00
Postgraduate	36	7.20
**Monthly income (AED)**		
≤10,000	287	57.40
11,000–50,000	76	15.20
≥51,000	8	1.60
Did not mention	129	25.80
**Employment status**		
Student	242	48.40
Housewife	75	15.00
Employed	157	31.40
Retired	2	0.40
Unemployed	24	4.80
**Employment status in health elated sector**		
^a^Healthcare student	53	15.63
^a^Non-healthcare student	286	84.37
^a^Healthcare worker	34	10.03
^a^Non-healthcare worker	305	90
**Civil status**		
Divorced	9	1.80
Married	185	37.00
Separated	7	1.40
Single	297	59.40
Widowed	2	0.40
**Comorbid**		
Hypertension	47	9.40
Diabetes	33	6.60
Dyslipidemia	31	6.20
Heart disease	17	3.40
Stroke	9	1.80
Other	41	8.20
No comorbid	254	50.80
Have heard about stroke	455	91.00
Know someone with a stroke	249	49.80
Know the risk factors for stroke	310	62.00
Know any warning signs of stroke	282	56.40
**Sources of information**		
Electronic media	252	50.40
Newspaper	54	10.80
TV	104	20.80
Radio	22	4.40
Friends	202	40.40
Healthcare provider	150	30.00

^a^ The denominator is 339.

### Knowledge on stroke

The mean (SD) knowledge score was 13.66 (5.31) and ranged from 2 to 24. Table 2 presents the responses regarding knowledge of stroke. Around (82%) of the study participants knew the brain was the organ affected by stroke, 41.8% knew that a stroke could be recurrent, and 63.2% knew that a stroke could be prevented. On the other hand, many participants (83.2%) knew that stroke affects the patients’ daily lives and activities.

**Table 2.** Participant responses to questions on stroke knowledge (n = 500).

	Response = yes N (%)
What organ of the body is affected by stroke? Brain	413 (82.60)
Stroke is preventable	316 (63.20)
A person can have a stroke more than once	209 (41.80)
Stroke affects daily activities	416 (83.20)
Stroke is preventable if treated early	373 (74.60)

**Figure 1.**
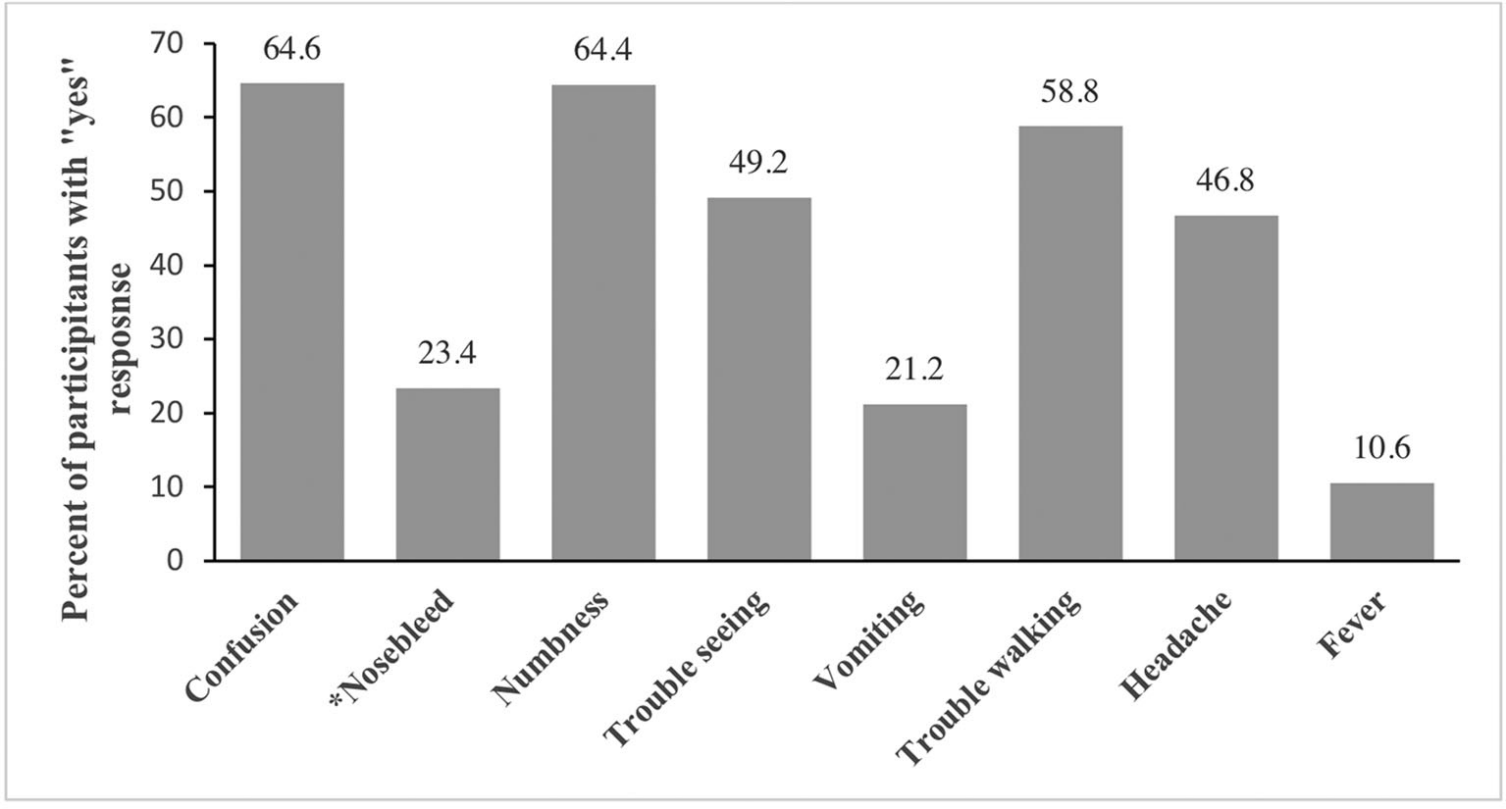
Distribution of knowledge of stroke signs and symptoms among survey participants (n = 500). The Y axis represents percentages of positive (yes) responses. (*) on x-axis label indicate incorrect sign/symptom of stroke.

Figure 1 presents positive (yes) responses to questions on the signs and symptoms of a stroke. More than half of the participants correctly identified confusion (64.6%), numbness (64.4%), and trouble walking (58.8%). Less than half could identify trouble seeing (49.2%), vomiting (21.2%), headache (46.8%), and fever (10.6%) as signs of a stroke. Approximately (23%) of the participants incorrectly identified a nose bleed as a sign of stroke.

**Figure 2.**
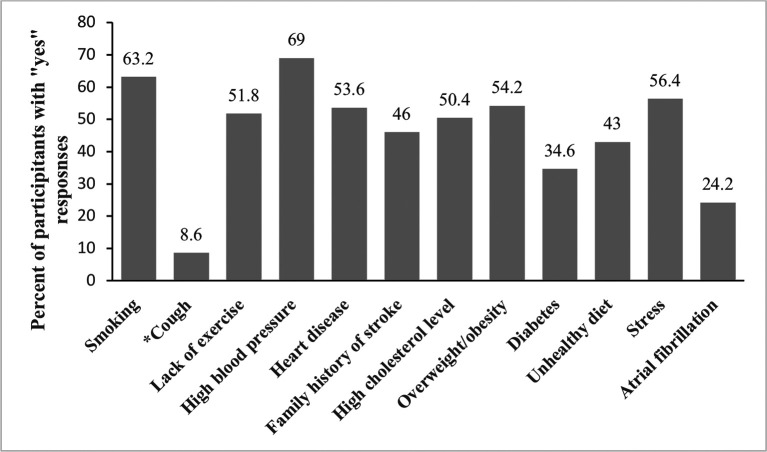
Distribution of knowledge of stroke risk factors among survey participants (n = 500). The Y axis represents percentages of positive (yes) responses. (*) on x-axis label indicates incorrect risk factor of stroke.

Figure 2 presents positive (yes) responses to questions on stroke risk factors. More than half of the participants correctly identified smoking (63.2%), lack of exercise (51.8%), high blood pressure (69%), heart disease (53.6%), high cholesterol (50.4%), being overweight/obese (54.2%), and stress (56.4%). Fewer than half knew about a family history of stroke (46%), diabetes (34.6%), an unhealthy diet (43 %), atrial fibrillation (24.2%), and 8% incorrectly identified cough as a risk factor for stroke.

### Attitude towards stroke

The mean (SD) score of participants’ attitudes towards stroke was 4.41 (1.40) and ranged from 1 to 6. Table 3 presents the participants’ attitudes toward stroke development in a person. Over two-thirds (70.2%) of the study participants would call an ambulance if someone showed signs and symptoms of stroke, (12.4%) would take the person to a hospital, and (4.6%) would call a healthcare provider.

**Table 3.** Participant responses to questions on attitudes toward stroke (n = 500).

	Response = yes N (%)
Do you think stroke requires prompt treatment?	436 (87.20)
**If someone shows signs and symptoms of stroke, what do you think you should do first?**	
Give them aspirin	59 (11.80)
Contact his/her family	5 (1.00)
Call an ambulance	351 (70.20)
Take them to the hospital or clinic	62 (12.40)
Call a health care provider	23 (4.60)
**Stroke prevention**	
A controlled diet in elderly individuals can prevent stroke	335 (67.00)
Strokes can be prevented by controlling blood pressure	360 (72.00)
Strokes can be prevented by controlling blood glucose levels	273 (54.60)
Strokes cannot be prevented	51 (10.20)

Regarding stroke prevention, more than two-thirds (67.0%) knew a controlled diet could prevent stroke, many (72.0%) knew control of hypertension was important, and over half (54.6%) knew the importance of the control of blood glucose. Merely 10.2% thought that stroke could not be prevented.

### Determinants of stroke knowledge and attitudes

We determined the variables associated with the knowledge and attitudes toward stroke using linear regression analysis. Note that the nine respondents with history of stroke were excluded from analysis and hence the sample size is 491. As shown in Table 4, a monthly income 11,000 to 50,000 AED (estimate = 0.52; P = 0.05) and being a student (estimate = 2.45; P = 0.04) were associated with positive knowledge. On the other hand, not having heard of stroke (estimate = -3.45; P < 0.01), not knowing someone with stroke (estimate = -2.03; P < 0.01), not receiving information from a healthcare provider (estimate = -1.35; P < 0.01), and lack of information on electronic media (estimate = -1.11; P = 0.02) were associated with worse knowledge ( Table 4).

**Table 4.** Association of participants’ characteristics with stroke knowledge (n = 491).

Variable	Parameter estimate/coefficient	Standard error	P-value
Non-healthcare student	0.31	1.49	0.83
Healthcare student	Ref		
Non-healthcare worker	-1.05	1.01	0.30
Healthcare worker	Ref		
Female	0.48	0.57	0.40
Age, years			
18–25	0.87	4.20	0.84
26–35	0.30	4.14	0.94
36–45	-0.17	4.11	0.97
46–55	0.58	4.11	0.89
56–65	0.14	4.57	0.98
65 and above	Ref		
Graduate	0.60	0.7	0.41
No formal education	-4.15	3.46	0.23
Postgraduate	0.55	1.02	0.59
Undergraduate	Ref		
Monthly income ≤10,000	0.52	0.66	0.43
11,000–50,000	1.76	0.89	0.05 *
≥51,000	1.37	1.97	0.49
Preferred not to mention the amount	Ref		
Employed	0.91	1.13	0.42
Housewife	2.02	1.24	0.10
Retired	-3.07	4.28	0.47
Student	2.45	1.20	0.04
Unemployed	Ref		
Hypertension, No	1.58	0.93	0.09
Diabetes, No	0.01	1.20	0.99
Dyslipidemia, No	1.87	1.11	0.09
Heart diseases, No	0.39	0.44	0.38
Another disease, No	1.53	1.24	0.22
No comorbid	1.69	1.08	0.12
Have you heard about stroke, No	-3.45	0.81	<0.01 **
Do you know anyone with a stroke, No	-2.03	0.47	<0.01 **
Healthcare provider, No	-1.35	0.54	0.01 **
Friends, No	0.64	0.45	0.16
Radio, No	-0.75	1.13	0.51
TV, No	-0.26	0.588	0.67
Newspapers, No	0.25	0.74	0.7
Electronic media, No	-1.11	0.45	0.02 *

N = 491 instead of 500 as we excluded people with a history of stroke for the regression analysis.

* P-value ≤ 0.05.

** P-value ≤ 0.01.

Regarding attitudes, being a non-healthcare worker (estimate = -0.73; P < 0.01) and lack of information on electronic media (estimate = -0.30; P = 0.02) were associated with poorer attitudes ( Table 5).

**Table 5.** Association of participants’ characteristics with stroke attitude (n = 491).

Variable	Parameter estimate/coefficient	Standard error	P-value
Non-healthcare student	0.04	0.42	0.92
Healthcare student	Ref		
Non-healthcare worker	-0.73	0.28	<0.01 **
healthcare worker	Ref		
Female	0.30	1.17	0.72
Age, years			
18–25	-0.42	1.16	0.97
26–35	-0.05	1.15	0.96
36–45	-0.06	1.15	0.68
46–55	0.48	1.28	0.63
56–65	0.61	1.17	0.72
65 and above	Ref		
Graduate	-0.15	0.20	0.48
No formal education	0.56	0.99	0.56
Postgraduate	-0.19	0.29	0.50
Undergraduate	Ref		
Monthly income ≤10,000	-0.09	0.18	0.62
11,000–50,000	0.33	0.25	0.18
≥51,000	-0.17	0.55	0.76
Preferred not to mention the amount	Ref		
Employed	-0.11	0.32	0.72
Housewife	0.03	0.35	0.93
Retired	-0.30	1.20	0.81
Student	0.12	0.33	0.72
Unemployed	Ref		
Hypertension, No	0.08	0.26	0.75
Diabetes, No	0.08	0.34	0.79
Dyslipidemia, No	0.43	0.31	0.16
Heart diseases, No	0.38	0.44	0.38
Another disease, No	0.25	0.35	0.48
No comorbid	0.20	0.30	0.50
Have you heard about stroke, No	-0.35	0.23	0.12
Do you know anyone with a stroke, No	-0.14	0.13	0.29
Healthcare provider, No	-0.31	0.15	0.03 *
Friends, No	-0.03	0.13	0.81
Radio, No	0.06	0.32	0.85
TV, No	0.06	0.16	0.70
Newspapers, No	0.02	0.21	0.92
Electronic media, No	-0.30	0.13	0.02 *

N = 491 instead of 500 as we excluded people with a history of stroke for the regression analysis.

* P-value ≤ 0.05.

** P-value ≤ 0.01.

## Discussion

The present study was conducted to assess the knowledge and attitudes towards stroke in a general population sample in the UAE during the COVID-19 pandemic. Generally, we identified suboptimal knowledge and attitudes toward stroke. Attitudes toward stroke prevention seemed suboptimal for preventive measures; many knew about hypertension, and less than two-thirds knew about blood glucose control. The sources of information were also varied and underutilized, especially healthcare providers.

Generally, and to the best of our knowledge, studies from the Middle East have reported suboptimal levels of knowledge of stroke. Most of the studies from the Middle East have reported inadequate knowledge regarding stroke risk factors and warning symptoms ^25^^–^^30^; on the other hand, only a few studies have reported an adequate level of awareness. ^31^^,^^32^ Similarly, studies from different parts of the world have reported an inadequate understanding of stroke in the general population. ^33^^,^^34^

More than half of our study participants were aged 18 to 25. There have been few previous studies on stroke awareness among adolescents and young adults. Studies from Nepal have reported knowledge of stroke in the younger demographics. ^35^^,^^36^ Participants reported knowing someone with a stroke which could have contributed to better understanding. ^36^ However, many participants did not recognize stroke as a brain disease. ^36^ Similarly, some studies in western countries have shown inconsistent awareness of this aspect. ^37^^,^^38^ In the Nepalese studies, many identified hypertension, alcohol, and smoking as risk factors. However, few could identify all risk factors together. Many believed that stroke could present with sudden weakness or numbness of limbs, and less than half were able to identify three or more symptoms of a stroke. More than two-thirds of participants believed stroke could be treated, and more than four-fifth believed stroke could be prevented. ^36^ Many said they would take patients to the hospital and that they would need immediate medical treatment.

Pradhan *et al.* reported better knowledge among male participants ^35^; however, Thapa *et al.* reported that gender was not associated with knowledge of risk factors or warning signs. ^36^ This was in contrast to our study, as gender was not a determinant of either knowledge or attitudes. Nevertheless, several studies have reported differences in knowledge scores by gender; females possessed better knowledge ^39^^–^^41^ which perhaps could be related to the fact that women experience more strokes ^42^ or knew someone with a stroke in the capacity of a caregiver. ^43^ Furthermore, a review has reported better knowledge of stroke warning signs in women compared with men ^44^; women tended to know more evidence-based stroke risk factors than men, which could be attributed to a more proactive health-seeking approach in women; stroke knowledge also appeared to be related to the country of study origin, age, education, and medical history.

In comparison, a recent study from the USA among adolescents reported that stroke knowledge was relatively inadequate. ^45^ Approximately half knew that stroke occurs in the brain, two-thirds said they would call emergency services, and about half were aware of the acronym FAST (face, arms, speech, time). The knowledge of stroke symptoms and risk factors was generally low, with no difference in scores according to gender in similarity to our findings. Furthermore, a surrogate marker of socioeconomic status, the parental education level, was used to assess the contribution to stroke knowledge; no relationship between survey scores and the father’s level of education was seen, but there was a significant association between survey scores and the mother’s level of education.

A community-based study from India, where stroke is a leading cause of morbidity and mortality, reported that participants knew the basic connotations of stroke and paralysis. However, knowledge about red flags and stroke risk factors was inadequate. ^34^ Signs and symptoms identified were paralysis and loss of consciousness, but there was a lack of awareness of headache, vomiting, and fits. Participants were well aware of hypertension as a risk factor but less for diabetes and smoking. Hypertension was one of the most frequently recognized risk factors, as in another study from Iran. ^45^ In comparison, a survey in South Korea reported better awareness about stroke; hypertension was the most common risk factor identified, and paresis was the most commonly reported symptom. Around two-thirds were able to identify one or more symptoms ^46^; in contrast, in our study, half of the participants identified five symptoms (results not tabulated).

In our study, knowledge and attitudes toward stroke did not differ by age category. Nevertheless, previous studies have consistently shown that different age groups were differently associated with knowledge and attitudes towards stroke. ^28^^,^^45^^,^^47^^,^^48^ In our study, being a student was associated with positive knowledge scores and a non-healthcare worker with poorer attitudes. Similar to our findings, a study from Saudi did not report that attitudes differed by gender. ^49^ Moreover, some studies have reported that knowledge differed with academic level, and higher education was a predictor of better knowledge. ^45^^,^^50^^–^^52^ Similarly, a recent European review reported that a higher socioeconomic position was associated with better knowledge of stroke risk factors and warning signs. ^53^ A review from the UK reported a good awareness of red flags of unilateral weakness and speech disturbance; however, the first point of contact mentioned was a general practitioner rather than emergency services. ^54^ In our study, a little over two-thirds said they would call an ambulance, and this finding is not very different from the Middle East. ^30^

It is reassuring to see that despite studies reporting inadequate understanding of stroke, there was a positive attitude toward calling an emergency in case a person displayed signs or symptoms of stroke, ^55^^,^^56^ while others are reporting taking the patient to the hospital. ^57^ In our study, being a non-health care worker was associated with poorer attitudes. Hence efforts should be made to reach out to different sectors and address gaps in knowledge and attitudes towards stroke. Moreover, electronic media campaigns could play an important role in raising public awareness and improving attitudes by employing a variety of social media platforms and types of messages.

Some of the strengths of this study are as follows: First, the study is one of the few studies that comprehensively quantify knowledge and practices and would help identify common knowledge gaps in the UAE population. Second, the study was conducted on a representative sample of the general population comprising of diverse backgrounds and provided a projection of the knowledge and attitudes in the community. Third, we used a validated and reliable questionnaire to collect responses. Lastly, we had a large sample size and a reasonable response rate, allowing us to conduct analyses with good statistical power to detect associations.

There are some limitations, however: The self-reported nature of specific measures such as income may lead to misclassification bias of the independent variables in the study. Moreover, self-reporting of information may be biased by overestimating or underestimating actual attitudes related to stroke. Next, as with studies of observational nature, it can be challenging to draw definite conclusions about causality and temporal relationships; hence we need further research with more robust study designs and pre-post interventional studies to assess the impact on knowledge and attitudes towards stroke in the community. Also, some of the respondents did not answer the questions if they were either working or studying in the healthcare field or not. Finally, the age distribution of participants in our study does not reflect the age distributed in the general population in the UAE. Bias in data collection may produce this problem. Nonetheless, we believe that the impact of this bias on the validity of the findings may not be significant.

## Conclusions

The present study showed a general inadequacy of knowledge regarding stroke and suboptimal attitudes towards someone presenting with stroke signs and symptoms. The community should be familiarized with the “FAST” acronym to recognize a stroke and access appropriate services as soon as possible. There is an urgent need for widespread educational interventions regarding stroke risk factors, especially among non-medical professions, and involving healthcare providers to address the growing burden of stroke worldwide, especially in the era of Covid-19, which increases the risk of stroke via infection and post-vaccination.

### Contribution to the field statement

The burden of stroke and its associated DALYs necessitates the evaluation of stroke KAP in the community. Especially in the Middle East, where despite the advances in stroke management, we continue to see a substantial stroke burden. As suggested by previous literature, knowledge and attitudes towards stroke may influence stroke prevention and outcome, and is a cornerstone of the WHO’s efforts toward increasing chronic disease literacy. In the UAE, literature is scarce about the level of knowledge of stroke in the community. Currently, this assessment is of outmost importance given the association of COIVD infection with stroke. Therefore, we aimed to conduct this study in a representative sample of the general population. Our study revealed suboptimal knowledge and suboptimal attitudes towards stroke, consistent with some studies in other parts of the region. We recommend that such findings be the base for educational awareness efforts among the general population and high-risk individuals in the community. This could improve stroke outcomes and encourage the adoption of healthy behaviors in all risk profile groups.

## Data Availability

Open Science Framework: Stroke Study, https://doi.org/10.17605/OSF.IO/5WNAF. ^58^
- Stroke study Responses and Scores.xlsx Open Science Framework: Stroke Study, https://doi.org/10.17605/OSF.IO/5WNAF. ^58^
- Stroke questionaire.docx -
STROBE_checklist_cross-sectional- Stroke Study.docx Data are available under the terms of the Creative Commons Zero “No rights reserved” data waiver (CC0 1.0 Public domain dedication).
