# Supplementary material for: Assessment of Knowledge and attitude towards Stroke among the UAE population during the COVID-19 pandemic: A cross-sectional study
Source: F1000Res. 2023 Oct 13;12:322. Originally published 2023 Mar 23. [Version 2] doi: 10.12688/f1000research.129873.2 (PMC10579853; doi:10.12688/f1000research.129873.2)
Supplement: Peer review history

# Reviewer report: Reviewer response for version 2

**DOI:** 10.5256/f1000research.157356.r215153

**Published:** 2023-11-03

**Authors:** Subish Palaian

**Affiliations:** 1 Department of Clinical Sciences, College of Pharmacy and Health Sciences, Ajman University, Ajman,, United Arab Emirates

Please accept the current version of the manuscript. IT can be now changed to "approved" from my side.

Is the work clearly and accurately presented and does it cite the current literature?

Yes

If applicable, is the statistical analysis and its interpretation appropriate?

Yes

Are all the source data underlying the results available to ensure full reproducibility?

Yes

Is the study design appropriate and is the work technically sound?

Yes

Are the conclusions drawn adequately supported by the results?

Partly

Are sufficient details of methods and analysis provided to allow replication by others?

Yes

Reviewer Expertise:

NA

I confirm that I have read this submission and believe that I have an appropriate level of expertise to confirm that it is of an acceptable scientific standard.

# Reviewer report: Reviewer response for version 2

**DOI:** 10.5256/f1000research.157356.r215154

**Published:** 2023-10-16

**Authors:** Adrià Arboix

**Affiliations:** 1 Cerebrovascular Division, Department of Neurology, University of Barcelona, Barcelona, Spain

I appreciate the authors efforts to include my comments. I do not have further comments.

Is the work clearly and accurately presented and does it cite the current literature?

Yes

If applicable, is the statistical analysis and its interpretation appropriate?

Yes

Are all the source data underlying the results available to ensure full reproducibility?

Yes

Is the study design appropriate and is the work technically sound?

Yes

Are the conclusions drawn adequately supported by the results?

Yes

Are sufficient details of methods and analysis provided to allow replication by others?

Yes

Reviewer Expertise:

Cerebrovascular diseases; stroke; lacunar infarcts, vascular cognitiva impairment.

I confirm that I have read this submission and believe that I have an appropriate level of expertise to confirm that it is of an acceptable scientific standard.

# Reviewer report: Reviewer response for version 2

**DOI:** 10.5256/f1000research.157356.r215155

**Published:** 2023-10-16

**Authors:** Walid Al-Qerem

**Affiliations:** 1 Department of Pharmacy, Faculty of Pharmacy, Al-Zaytoonah University of Jordan, Amman, Amman Governorate, Jordan

The manuscript is ready for indexing in its present form

Is the work clearly and accurately presented and does it cite the current literature?

Yes

If applicable, is the statistical analysis and its interpretation appropriate?

Yes

Are all the source data underlying the results available to ensure full reproducibility?

Yes

Is the study design appropriate and is the work technically sound?

Yes

Are the conclusions drawn adequately supported by the results?

Yes

Are sufficient details of methods and analysis provided to allow replication by others?

Yes

Reviewer Expertise:

Therapy

I confirm that I have read this submission and believe that I have an appropriate level of expertise to confirm that it is of an acceptable scientific standard.

# Reviewer report: Reviewer response for version 1

**DOI:** 10.5256/f1000research.142590.r178418

**Published:** 2023-09-25

**Authors:** Subish Palaian

**Affiliations:** 1 Department of Clinical Sciences, College of Pharmacy and Health Sciences, Ajman University, Ajman,, United Arab Emirates

I appreciate the authors for undertaking this important piece of research. It is a well-conducted study with scientific merits. This study can be a baseline research for many potential future researchers and hence must be published.

 Title: The title should be made simple and clearer. Currently, it is too long and confusing.

 Keywords: Need to be alphabetically arranged

 Stroke is generally considered to occur in elderly people. However, in this research, the participants are mainly young people/students. Authors need to mention this as a major limitation. This limits the study to generalize the findings to the general public. It would have been nice to focus only on the students rather than the general public in this case.

 The Introduction section has to mention more about the probable mechanism of stroke in COVID-19 patients. Authors need to focus more on the topic with more literature added.

 It is not clear why authors have chosen ‘hypertension’ to assume the prevalence of stroke. This needs more explanation.

 The questionnaire design is not clearly mentioned in the methodology section. The construct, content, and face validity need to be mentioned in detail. The final questionnaire must be added as an appendix. The authors also need to mention why they developed a new questionnaire rather than adopting an existing one. What is the uniqueness of the newly developed tool? The actual Cronbach alpha value has to be mentioned. The data collection process has to be elaborated. What was the mode of circulation of the questionnaire? How were the subjects chosen?

 The discussion rarely mentions about the relationship between COVID-19 and stroke knowledge. Hence some rewriting is needed.

 The Conclusion is misleading. How can the authors say the knowledge on stroke is ‘inadequate’ and ‘suboptimal’ attitudes? Authors need to first establish the criteria for how they arrived at such conclusions. This has to be clearly mentioned in the methodology.

Is the work clearly and accurately presented and does it cite the current literature?

Yes

If applicable, is the statistical analysis and its interpretation appropriate?

Yes

Are all the source data underlying the results available to ensure full reproducibility?

Yes

Is the study design appropriate and is the work technically sound?

Yes

Are the conclusions drawn adequately supported by the results?

Partly

Are sufficient details of methods and analysis provided to allow replication by others?

Yes

Reviewer Expertise:

Pharmacovigilance

I confirm that I have read this submission and believe that I have an appropriate level of expertise to confirm that it is of an acceptable scientific standard, however I have significant reservations, as outlined above.

# Response

**Published:** 2023-10-10

**Authors:** Azza Ramadan

**Affiliations:** Al Ain University, United Arab Emirates

Dear Reviewer,

 Thank you for taking the time to review our work titled **“**Are we aware of stroke risk factors and warning signs amid COVID-19? Knowledge and attitude towards risk-factors, signs and symptoms of stroke among the UAE population during COVID-19 pandemic: A cross-sectional study” ****and providing us with your insightful feedback to improve the manuscript. We have included answers in the manuscript to the questions put forth. Please see below the point-by-point responses to the questions.

**Q1. Title: The title should be made simple and clearer. Currently, it is too long and confusing.**

 Are we aware of stroke risk factors and warning signs amid COVID-19? Knowledge and attitude towards risk-factors, signs and symptoms of stroke among the UAE population during COVID-19 pandemic: A cross-sectional study

 Answer: Thank you for your comment. The above title was shortened to

 “Assessment of Knowledge and attitude towards Stroke among the UAE population during COVID-19 pandemic: A cross-sectional study”

**Q 2. Keywords: Need to be alphabetically arranged**

 Answer: Please see the re-arranged keywords that were inserted in the manuscript

 Attitude, Awareness, Cross-sectional, Knowledge, Stroke, Survey

**Q 3. Stroke is generally considered to occur in elderly people. However, in this research, the participants are mainly young people/students. Authors need to mention this as a major limitation. This limits the study to generalize the findings to the general public. It would have been nice to focus only on the students rather than the general public in this case.**

 Answer: The authors agree with the comment. The following is mentioned in the limitation section of the manuscript:

 “ the age distribution of participants in our study was mostly young people/students and hence does not reflect the age distributed in the general population in the UAE. Bias in data collection may produce this problem. Nonetheless, we believe that the impact of this bias on the validity of the findings may not be significant”

**Q 4. The Introduction section has to mention more about the probable mechanism of stroke in COVID-19 patients. Authors need to focus more on the topic with more literature added.**

 Answer: Thank you for the suggestion. The following was added to the introduction section.

 ACE receptors, where the COVID virus binds to gain intracellular entry, is expressed in many cells, including epithelial and endothelial cells, which trigger an immediate immunological activation that can lead to hypercoagulability and thrombosis. Multisystem thrombosis, including ischemic stroke, has been associated with severe COVID-19 infection. The pathogenesis is further compounded, given that the fibrinolytic pathway ceases operation. While the pathogenesis of COVID-19-related neurovascular events is not yet clear, some major pathogenic mechanisms have been put forth. These include innate system hyperinflammation, endothelial dysfunction, and disruption of the renin-angiotensin-aldosterone system, hence impacting blood flow, oxidative stress and excessive platelet aggregation (Wijeratne et al. 2021).

 Reference: Wijeratne T, Gillard Crewther S, Sales C, Karimi L. COVID-19 Pathophysiology Predicts That Ischemic Stroke Occurrence Is an Expectation, Not an Exception-A Systematic Review. *Front Neurol*. 2021;11:607221. Published 2021 Jan 28. doi:10.3389/fneur.2020.607221

**Q 5. It is not clear why authors have chosen ‘hypertension’ to assume the prevalence of stroke. This needs more explanation.**

 Answer: Thank you for this valuable comment.

 Hypertension is the single most important modifiable stroke risk factor therefore, its value from the literature was used in the calculation of the sample size. This approach has also been used in previous literature: https://www.tandfonline.com/doi/full/10.1080/2331205X.2017.1327129

 (Namaganda P, Nakibuuka J, Kaddumukasa M, Katabira E. Stroke in young adults, stroke types and risk factors: a case control study. *BMC Neurol*. 2022;22(1):335. Published 2022 Sep 6. doi:10.1186/s12883-022-02853-5)

**Q 6. The questionnaire design is not clearly mentioned in the methodology section. The construct, content, and face validity need to be mentioned in detail. The final questionnaire must be added as an appendix. The authors also need to mention why they developed a new questionnaire rather than adopting an existing one. What is the uniqueness of the newly developed tool? The actual Cronbach alpha value has to be mentioned. The data collection process has to be elaborated. What was the mode of circulation of the questionnaire? How were the subjects chosen?**

**A) The construct, content, and face validity need to be mentioned in detail**

 Answer: Thank you for this comment. The bolded text is the additional info added to the manuscript to clarify this point as requested.

**The survey started with a brief introduction that described the study objectives, emphasized the confidentiality of the participants, and informed them that completing the survey represents consent to participate in the study. The survey included socio-demographic questions, twenty-five questions evaluating the knowledge of stroke comprising the organ affected, stroke attributes (preventable or recurrent), effect on daily activities, treatment, prevention, risk factors, and signs and symptoms. Six questions evaluating attitude (the approach) towards preventative measures and actions to take if someone showed signs and symptoms of a stroke. All knowledge and attitude questions had a yes/no answer. Lastly, there was a question about the sources of information. For the 25-item knowledge questions, the score range was 0-25. For attitudes, the score range was 0-6. Each correct statement for knowledge and optimal attitude got a 1; otherwise, 0.**

 A questionnaire draft was written and sent to a panel of experts in the pharmacy profession at Al-Ain University **for face and content validity.** They examined many factors of the questionnaire, including the length, conciseness, language, clarity, time, appropriateness, and bias of questions. Content validation of a questionnaire was aligned with recommendations.

 The content validity was as follows:

**B) The final questionnaire must be added as an appendix.**

 Answer: Thank you for this comment. Please find the questionnaire at the extended data section:

 Open Science Framework: Stroke Study, https://doi.org/10.17605/OSF.IO/5WNAF. ^58^

 - Stroke questionaire.docx

**C) The authors also need to mention why they developed a new questionnaire rather than adopting an existing one. What is the uniqueness of the newly developed tool?**

 Answer: Thank you for this valuable comment. The yellow highlights is the additional info. that was added to the methods section (Design of questionnaire):

 The questionnaire was developed after a careful literature review of previous studies utilizing standardized and validated instruments (25–28). Some questions were included to suit the public within the UAE e.g. city of residence, place of birth.

 The uniqueness of this questionnaire is that it is designed to suit the UAE public. To the best of our knowledge and up to the time this study was conducted, there was not any single study that was conducted across the UAE.

**D) The actual Cronbach alpha value has to be mentioned.**

 Answer:  Thank you. The following was added

 The Cronbach alpha value determined was 0.72. According to Nunnally's criteria, an *α* *≥ 0.70* should be regarded as an acceptable reliability

**E) The data collection process has to be elaborated.**

 Answer: Thank you for this comment. The following was included:

 Data was collected from study participants using Google Forms between September 2021 to January 2022. Participants were briefed about the study’s purpose and informed about the study’s confidentiality and anonymity policy. Each participant was invited to answer the survey after consent. The questionnaire was self-administered.

**F) What was the mode of circulation of the questionnaire?**

 Answer: The following was added to the method sections

 The online, self-administered survey was randomly distributed via a convenience sampling technique

**G) How were the subjects chosen?**

 Answer: Thank you for your comment. Please refer to the Inclusion and exclusion criteria:

 They were the following:

 UAE residents (> 2 years living in the UAE) regardless of their nationality, over the age of 18 years and consented (on the questionnaire by ticking the agreement option) to participate were included in this study. Participants who lived < 2-years in the UAE or did not consent to participate were excluded.

**Q 7. The discussion rarely mentions about the relationship between COVID-19 and stroke knowledge. Hence some rewriting is needed.**

 Answer: Thank you for the feedback. The following will be inserted after 7 ^th^ paragraph of the discussion (highlighted yellow in the manuscript).

 We observed that inadequate knowledge was significantly associated with not having heard of stroke, not knowing someone with a stroke, and not receiving stroke-related information from either a healthcare provider or electronic media. Note that the study was conducted during the COVID-19 when there was a nationwide extended lockdown. Hence, the lack of stroke knowledge and awareness could be due to the public limited access to healthcare providers or due to the limited healthcare provider-led educational campaigns during the quarantine period. Also, the lack of use of electronic media by the participants could have contributed to the public’s gap in knowledge. Hence, overall, the lack of accessibility to healthcare providers and perhaps also to electronic media could have resulted in poor stroke awareness and knowledge. Stroke inadequate knowledge is of great concern given that ischemic stroke is a well-documented side effect of COVID-19. In a US study, it was observed that 46.35% of imaging confirmed ischemic stroke patients had COVID-19 (Belani et al. 2020). Also,  the incidence of stroke in COVID-19 patients ranged between 0.9% to 3.3% in several large retrospective studies. Also, higher mortality was reported in patients with COVID-19 who have ischemic stroke compared to control ischemic stroke patients. Furthermore, ischemic stroke was shown to develop in COVID-19 patients with or without co-morbidities (Elfasi et al. 2021). Hence, stroke knowledge-based educational campaigns provided and led by health care personnel are of utmost importance during the pandemic.

 References

 - Belani P, Schefflein J, Kihira S, et al. COVID-19 Is an Independent Risk Factor for Acute Ischemic Stroke. *AJNR Am J Neuroradiol*. 2020;41(8):1361-1364. doi:10.3174/ajnr.A6650

 - Elfasi, A., Echevarria, F. D., Rodriguez, R., Roman Casul, Y. A., Khanna, A. Y., Mankowski, R. T., & Simpkins, A. N. (2021). Erratum to "Impact of COVID-19 on Future Ischemic Stroke Incidence" [eNeurologicalSci, 22C (2021) 100325]. *eNeurologicalSci*, *23*, 100342. https://doi.org/10.1016/j.ensci.2021.100342

**Q 8. The Conclusion is misleading. How can the authors say the knowledge on stroke is ‘inadequate’ and ‘suboptimal’ attitudes? Authors need to first establish the criteria for how they arrived at such conclusions. This has to be clearly mentioned in the methodology.**

 Answer: The following was placed in the methodology section-Design of the of questionnaire:

 Based on the modified Bloom’s cut-off point, a participant who scored ≥80% of the correct knowledge questions (≥20 points out of 25) was considered as having “good/adequate knowledge”; moderate if the score was between 60 and 79%, (≥15-19 out of 25), and poor/inadequate if the score was less than 60% (<15 points out of 25). For attitude, a respondent who scored ≥80% of the correct attitude questions (≥4.8 points out of 6) was considered as having a “good attitude” or moderate/suboptimal attitude if the score was between 60 and 79%, (≥3.6-4.7 out of 6), and poor attitude if the score was less than 60% (<3.6 points out of 6) (Feleke et al. 2021).

# Reviewer report: Reviewer response for version 1

**DOI:** 10.5256/f1000research.142590.r203646

**Published:** 2023-09-08

**Authors:** Adrià Arboix

**Affiliations:** 1 Cerebrovascular Division, Department of Neurology, University of Barcelona, Barcelona, Spain

The authors present the results of a cross-sectional study aimed to assess the general public knowledge and attitudes on stroke and stroke risk factors in the United Arab Emirates during the COVID-19 pandemic. 500 participants ≥ 18 years and from the general public in The United Arab Emirates completed a self-administered questionnaire constructed in Arabic and English on sociodemographic characteristics and stroke knowledge and attitudes. The authors identified poor knowledge and suboptimal attitudes toward stroke. For a better presentation of the data, it is necessary to clarify the following issues:

1. It would be interesting to know if there were differences between the young (18-25 years old) and non-young healthy recruits with respect to knowledge and attitudes toward stroke.
2. In the Discussion, it should be noted that in a study on the knowledge of stroke in Catalonia (Spain) it was observed a scant knowledge of cerebrovascular disease in a sample of teenagers. Therefore, this knowledge should be integrated into the school curriculum (see and add this reference Rev Neurol 2003; 37: 500).
3. A brief concluding comment on other possible lines of future research on the presented topic would be appreciated

Is the work clearly and accurately presented and does it cite the current literature?

Yes

If applicable, is the statistical analysis and its interpretation appropriate?

Yes

Are all the source data underlying the results available to ensure full reproducibility?

Yes

Is the study design appropriate and is the work technically sound?

Yes

Are the conclusions drawn adequately supported by the results?

Yes

Are sufficient details of methods and analysis provided to allow replication by others?

Yes

Reviewer Expertise:

Cerebrovascular diseases; stroke; lacunar infarcts, vascular cognitiva impairment.

I confirm that I have read this submission and believe that I have an appropriate level of expertise to confirm that it is of an acceptable scientific standard, however I have significant reservations, as outlined above.

## References

[1] : [Scant knowledge about cerebrovascular disease in a sample of teenagers]. *Rev Neurol* .37(5) :500 14533103

# Response

**Published:** 2023-10-10

**Authors:** Azza Ramadan

**Affiliations:** Al Ain University, United Arab Emirates

Dear Reviewer,

 Thank you for taking the time to review our work titled “Are we aware of stroke risk factors and warning signs amid COVID-19? Knowledge and attitude towards risk-factors, signs and symptoms of stroke among the UAE population during COVID-19 pandemic: A cross-sectional study” and providing us with your insightful feedback to improve the manuscript. We have included answers in the manuscript to the questions put forth. Please see below the point-by-point responses to the questions.

**1. It would be interesting to know if there were differences between the young (18-25 years old) and non-young healthy recruits with respect to knowledge and attitudes toward stroke**.

 Author Response: Thank you for your comment.  Indeed, our findings suggest that knowledge among young participants (18-25 years old) was not statistically different in comparison to the ³ 65 years old group (estimate coefficient= 0.87; P=0.84, Table 4). Similarly, there was not a statistical difference in attitude scores when comparing the two groups (estimate coefficient= -0.42; P=0.84, Table 5).

**2. In the Discussion, it should be noted that in a study on the knowledge of stroke in Catalonia (Spain) it was observed a scant knowledge of cerebrovascular disease in a sample of teenagers. Therefore, this knowledge should be integrated into the school curriculum (see and add this reference Rev Neurol 2003; 37: 500).**

 Author Response: Thank you for your comment.

 The following was added to the discussion section.

 Additionally, limited knowledge of cerebrovascular disease was observed among teenagers in Spain, hence the need for integrating topics related to neurovascular disease within the school curriculum (Arboix and Arboix-Alió, 2003).

 Reference: Arboix A, Arboix-Alió A, Hernández-Arrieta M. Escaso conocimiento de la enfermedad vascular cerebral en una muestra de jovenes adolescentes [Scant knowledge about cerebrovascular disease in a sample of teenagers]. Rev Neurol. 2003;37(5):500.

**3. A brief concluding comment on other possible lines of future research on the presented topic would be appreciated**

 Author Response: Thank you for the suggestion:

 The following was inserted at the end of the discussion section:

 The future direction of the study can focus on conducting a cross-sectional study to assess the stroke knowledge and attitude among COVID-19-infected individuals and COVID-19 vaccine recipients, a significant risk group highly susceptible to stroke occurrence. Also, it is interesting to asses if stroke incidence dropped post-COVID-19 period.

# Reviewer report: Reviewer response for version 1

**DOI:** 10.5256/f1000research.142590.r167530

**Published:** 2023-04-12

**Authors:** Walid Al-Qerem

**Affiliations:** 1 Department of Pharmacy, Faculty of Pharmacy, Al-Zaytoonah University of Jordan, Amman, Amman Governorate, Jordan

The current cross-sectional study highlights the level of knowledge and attitude of stroke and among a sample of the UAE population. Also, the association of variables including sociodemographics with knowledge and attitude scores was carried out using linear regression analysis. The rationale of the study is sound given the link between COVID 19 infection and vaccination with stroke. Results are clear and organized with acceptable statistical analysis. The conclusions drawn from the findings are appropriate and not overinterpreted. The study is of significance given the findings and the recommendations for educational awareness among the general population and high-risk individuals in the community. However, there is an issue with the population representation.

 The following recommendations to improve the paper are listed below:

**Abstract:**

**Conclusion section**

 “Presently, this is of utmost necessity, given the increased occurrence of stroke and its severity among COVID-19 patients.” Reword this sentence as it is unclear

                           

**Introduction:**

 1 ^st^ paragraph: Indicate what co-morbidities that can increase the risk of occurrence, given that the survey has questions regarding medical history of subjects.

 2 ^nd^ paragraph, last sentence: “Regarding stroke, early identification of symptoms and appropriate and timely management." Remove “and” from symptoms and appropriate

 3 ^rd^ paragraph, last sentence:

- “Analysis of reports pertaining to the COVID pandemic shed light etc…," insert had after the word pandemic
- Need to expand on why Covid increase stroke occurrence. Provide recent references  
- Elaborate on why Covid vaccine can cause stroke?

**Methods:**

- State the domains clearly
- What constitutes poor, moderate and good for knowledge/attitude scores?  
- What part of the population was the study performed on?
- What was/were the main attitude question(s)? Provide examples to focus the reader’s attention.

**Results:**

 (Table 5). There was no mention between the association of lack of information from health care provide with attitude in the results description

**Discussion:**

- Page 10, last paragraph: “This was in contrast to our study, as gender etc..” doesn’t fit the context. Revise the statement
- Knowledge was suboptimal, state plausible reasons for that. Is there a disconnect between resources and the population? Is there scarcity of provided information?
- ~ 30% of the UAE are bachelors graduates. However, post analysis, nearly half of the subjects were young and undergraduate students. Provide the implication regarding this discrepancy?
- What about rural based participants? Were they included in the study. This needs to be included in the limitation section

Is the work clearly and accurately presented and does it cite the current literature?

Yes

If applicable, is the statistical analysis and its interpretation appropriate?

Yes

Are all the source data underlying the results available to ensure full reproducibility?

Yes

Is the study design appropriate and is the work technically sound?

Yes

Are the conclusions drawn adequately supported by the results?

Yes

Are sufficient details of methods and analysis provided to allow replication by others?

Yes

Reviewer Expertise:

Therapy

I confirm that I have read this submission and believe that I have an appropriate level of expertise to confirm that it is of an acceptable scientific standard, however I have significant reservations, as outlined above.

# Response

**Published:** 2023-10-10

**Authors:** Azza Ramadan

**Affiliations:** Al Ain University, United Arab Emirates

Dear Reviewer,

 Thank you for taking the time to review our work titled **“ **Are we aware of stroke risk factors and warning signs amid COVID-19? Knowledge and attitude towards risk-factors, signs and symptoms of stroke among the UAE population during COVID-19 pandemic: A cross-sectional study**”****** and providing us with your insightful feedback to improve the manuscript. We have included answers in the manuscript to the questions put forth. Please see below the point-by-point responses to the questions.

**Abstract:**

**Conclusion section**

 “Presently, this is of utmost necessity, given the increased occurrence of stroke and its severity among COVID-19 patients.” Reword this sentence as it is unclear

 Response: Thank you for your comment. The sentence has been reworded to “Presently, this is of utmost necessity, given the increased occurrence of stroke and its severity during the COVID-19 pandemic ”

                           

**Introduction:**

 1) 1 ^st^ paragraph: Indicate what co-morbidities that can increase the risk of occurrence, given that the survey has questions regarding medical history of subjects.

 Response: Thank you for your comment. The following has been added to the end of the 1 ^st^ paragraph in the introduction section.

 "Also, the high prevalence of comorbidities such as hypertension, congenital heart disease, previous stroke, and diabetes is associated with increased risk for all types of stroke (Zhang et al. 2021, Elamy et al. 2020)"

 References

 - Zhang, Y., Wang, C., Liu, D. et al. Association of total pre-existing comorbidities with stroke risk: a large-scale community-based cohort study from China. BMC Public Health 21, 1910 (2021).

 - Elamy, A., Shuaib, A., Carriere, K., & Jeerakathil, T. (2020). Common Comorbidities of Stroke in the Canadian Population. Canadian Journal of Neurological Sciences, 47(3), 314-319. doi:10.1017/cjn.2020.17

 2) 2 ^nd^ paragraph, last sentence: “Regarding stroke, early identification of symptoms and appropriate and timely management." Remove “and” from symptoms and appropriate

 Response: The word “and” has been removed. The sentence now in the manuscript is: “Regarding stroke, early identification of symptoms, appropriate and timely management.

 3) 3 ^rd^ paragraph, last sentence:

- “Analysis of reports pertaining to the COVID pandemic shed light etc…," insert had after the word pandemic
- Response: “Analysis of reports pertaining to the COVID pandemic had shed light, etc….

4) Need to expand on why COVID increase stroke occurrence. Provide recent references

 Response. Thank you for the comment. The following was added to the intro section: Multisystem thrombosis, including ischemic stroke, has been associated with severe COVID-19 infection. The pathogenesis is further compounded, given that the fibrinolytic pathway ceases operation. Also, ACE receptors, where the COVID-19 virus binds to gain intracellular entry, are expressed in many cells, including endothelial cells of the blood vessels. This could disrupt the renin–angiotensin system, which modulates blood flow, further contributing to the pathogenesis process (Wijeratne et al. 2021).

 Response: Wijeratne, T., Gillard Crewther, S., Sales, C., & Karimi, L. (2021). COVID-19 Pathophysiology Predicts That Ischemic Stroke Occurrence Is an Expectation, Not an Exception-A Systematic Review. Frontiers in neurology, 11, 607221. https://doi.org/10.3389/fneur.2020.607221

 5) Elaborate on why the COVID vaccine can cause stroke.

 Response: The vaccine's most adverse side effect is vaccine-induced immune thrombocytopenia and thrombosis (VITT), also known as thrombosis with thrombocytopenia syndrome. This immune-mediated condition is caused by the development of pathological anti-platelet factor 4 (PF4) antibodies following vaccination against COVID-19, which leads to intense activation of platelets and the coagulation system. The subsequent clinical syndrome includes life-threatening thrombosis and secondary haemorrhage (Kolahchi et al. 2022).

 Reference:  Kolahchi, Z., Khanmirzaei, M., & Mowla, A. (2022). Acute ischemic stroke and vaccine-induced immune thrombotic thrombocytopenia post COVID-19 vaccination; a systematic review. Journal of the neurological sciences, 439, 120327. https://doi.org/10.1016/j.jns.2022.120327

**6) Methods:**

- State the domains clearly
- Response: Thank you for this comment. Below is the additional information added to the manuscript to clarify the domains.
- The survey started with a brief introduction that described the study objectives, emphasized the confidentiality of the participants, and informed them that completing the survey represents consent to participate in the study. The survey included the following domains: socio-demographic questions, twenty-five questions evaluating the knowledge of stroke comprising the organ affected, stroke attributes (preventable or recurrent), effect on daily activities, treatment, prevention, risk factors, and signs and symptoms. Six questions evaluating attitude (the approach) towards preventative measures and actions to take if someone showed signs and symptoms of a stroke. All knowledge and attitude questions had a yes/no answer. Lastly, there was a question about the sources of information. For the 25-item knowledge questions, the score range was 0-25. For attitudes, the score range was 0-6. Each correct statement for knowledge and optimal attitude got a 1; otherwise, 0.

 

- What constitutes poor, moderate and good for knowledge/attitude scores?  
- Response: The following was placed in the methodology section of the questionnaire: Based on the modified Bloom’s cut-off point, a participant who scored ≥80% of the correct knowledge questions (≥20 points out of 25) was considered as having “good/adequate knowledge”; moderate if the score was between 60 and 79%, (≥15-19 out of 25), and poor/inadequate if the score was less than 60% (<15 points out of 25). For attitude, a respondent who scored ≥80% of the correct attitude questions (≥4.8 points out of 6) was considered as having a “good attitude”, moderate/suboptimal attitude if the score was between 60 and 79%, (≥3.6-4.7 out of 6), and poor attitude if the score was less than 60% (<3.6 points out of 6) (Feleke et al. 2021).

 

- What part of the population was the study performed on?
- Response: The study included both locals (Emiratis) and expats (residents)

 

- What was/were the main attitude question(s)? Provide examples to focus the reader’s attention.
- Response: Six questions evaluating attitude (the approach) towards preventative measures and actions to take if someone shows signs and symptoms of a stroke.

**7) Results:**

 (Table 5). There was no mention between the association of lack of information from health care provide with attitude in the results description

 Response: Thank you for the comment. The following (in yellow highlight) has been added:

  “Regarding attitudes, being a non-healthcare worker (estimate = -0.73; P < 0.01) and lack of information from health care provider (estimate = -0.31; P=0.03) and on electronic media (estimate = -0.30; P = 0.02) were associated with poorer attitudes

**8) Discussion:**

- Page 10, last paragraph: “This was in contrast to our study, as gender etc..” doesn’t fit the context. Revise the statement
- Response: Thank you. Replaced the above with “Similar to Thapa et al. study (36), in our study, gender...............

 

- Knowledge was suboptimal; state plausible reasons for that. Is there a disconnect between resources and the population? Is there a scarcity of provided information?
- Response: Thank you for the feedback. The following have been added to the discussion section: We observed that poor knowledge was significantly associated with not having heard of stroke, not knowing someone with a stroke, and not receiving stroke-related information from either a healthcare provider or electronic media. Note that the study was conducted during COVID-19 when there was a nationwide extended lockdown. Hence, the lack of stroke knowledge and awareness could be due to the public limited access to healthcare providers or limited healthcare provider-led educational campaigns during quarantine. Also, the lack of use of electronic media by the participants could have contributed to the public’s gap in knowledge. Hence, the lack of accessibility to healthcare providers and perhaps also to electronic media could have resulted in poor stroke awareness and knowledge.

 

- ~ 30% of the UAE are bachelor's graduates. However, post-analysis, nearly half of the subjects were young and undergraduate students. Provide the implication regarding this discrepancy?
- Response: The authors agree with the comment. The following is mentioned in the limitation section of the manuscript: “ the age distribution of participants in our study was mostly young people/students and hence does not reflect the age distributed in the general population in the UAE. Bias in data collection may produce this problem. Nonetheless, we believe that the impact of this bias on the validity of the findings may not be significant”

 

- What about rural-based participants? Were they included in the study? This needs to be included in the limitation section
- Response: The following has been added to the limitation section: “While the study was conducted in several UAE major urban cities, rural cities participants were not represented.”
